# Comparison of Four Universal Adhesive Protocols for the Internal Adaptation of Composite Restorations in Primary Teeth: An In Vitro Micro-CT Study

**DOI:** 10.3390/ma19163488

**Published:** 2026-08-18

**Authors:** Elif Aslı Demir, Akif Demirel, Arda Büyüksungur

**Affiliations:** 1 Department of Pediatric Dentistry, Faculty of Dentistry, Ankara University, 06560 Ankara, Turkey; demirea@ankara.edu.tr; 2 Department of Basic Medical Sciences, Faculty of Dentistry, Ankara University, 06560 Ankara, Turkey; abuyuksungur@ankara.edu.tr

**Keywords:** universal adhesive, primary teeth, internal adaptation, composite restoration, micro-computed tomography, self-etch, selective enamel etching

## Abstract

**Highlights:**

- Internal adaptation of Class V composite resin restorations in primary second molars was evaluated using micro-computed tomography.
- An overall difference in whole-cavity interfacial gap volume percentage was detected among the four tested adhesive protocols.
- After multiplicity adjustment, a significant pairwise difference was detected only between the two G2-BOND Universal groups.
- G2-BOND Universal used in the self-etch mode showed a lower whole-cavity interfacial gap volume percentage than when used with selective enamel etching.
- No other adjusted pairwise comparison reached statistical significance.

**Abstract:**

Achieving an acceptable adhesive interface is necessary for the long-term success of composite restorations in primary teeth. This study compared the three-dimensional internal adaptation of composite restorations in primary second molars among four prespecified universal adhesive product–application protocol combinations using micro-computed tomography (micro-CT). Seventy-six extracted primary second molars were randomly allocated to four experimental groups (*n* = 19): G2-BOND Universal/self-etch, G2-BOND Universal/selective enamel etching, G-Premio Bond/self-etch, and G-Premio Bond/selective enamel etching. Following restoration and thermocycling (5000 cycles), whole-cavity interfacial gap volume percentages were quantified using micro-CT. Data were analyzed using the Kruskal–Wallis test followed by Dunn–Bonferroni-adjusted pairwise comparisons. An overall difference was detected among the four experimental groups (*p* = 0.016). Following adjustment for multiple comparisons, the G2-BOND Universal self-etch group showed a lower whole-cavity interfacial gap volume percentage than the corresponding selective enamel-etching group (adjusted *p* = 0.013). No other adjusted pairwise comparison reached statistical significance. Within the limitations of this laboratory study, these findings are restricted to the tested product–application protocol combinations and do not demonstrate an interaction between adhesive system and application mode.

## 1. Introduction

Achieving a durable adhesive interface remains one of the greatest challenges in contemporary restorative and pediatric dentistry [1,2,3]. Although remarkable advances have been made in adhesive materials and clinical protocols, degradation of the resin–tooth interface continues to compromise the longevity of resin composite restorations [4,5,6]. This issue is particularly relevant in pediatric dentistry, where resin composites are increasingly used to restore primary teeth and maintain the primary dentition until physiological exfoliation. Consequently, optimizing adhesive performance has become a major focus of restorative research, especially considering the unique structural characteristics of primary dental tissues [3,7,8].

The introduction of universal adhesives has simplified adhesive procedures by allowing clinicians to use a single material with self-etch, selective enamel etching or etch-and-rinse strategies according to the clinical situation [1,2,9,10,11]. Their simplified application protocols are particularly attractive in pediatric dentistry, where reduced chairside time and easier moisture control may improve treatment efficiency and patient cooperation [3,7,8]. Nevertheless, adhesive performance may vary among specific adhesive product–application protocol combinations. Systematic reviews have consistently demonstrated that phosphoric acid etching generally enhances enamel bond strength, whereas its effect on dentin bonding remains less consistent and appears to depend on the specific adhesive formulation. Consequently, there is still no consensus regarding the optimal application strategy for all universal adhesive systems [2,9,11,12].

Evidence regarding adhesive performance in primary teeth is considerably more limited than that available for permanent dentition. Primary teeth differ from permanent teeth in several structural characteristics relevant to adhesion. Primary enamel and dentin are thinner and generally less mineralized, primary enamel commonly presents a relatively prominent superficial aprismatic layer, and primary dentin exhibits differences in tubular density and morphology. Consequently, bonding behavior and adhesive protocols developed for permanent teeth cannot be directly extrapolated to primary teeth [3,7,8,13,14]. Furthermore, most published studies have evaluated universal adhesives using bond strength tests or microleakage assessments, whereas relatively few have investigated three-dimensional internal adaptation [1,9,10,11]. Although bond strength and internal adaptation are closely related, they reflect different aspects of adhesive performance. While bond strength evaluates the mechanical resistance of the adhesive interface, internal adaptation provides volumetric information regarding interfacial gap formation throughout the entire restoration and may therefore offer additional insight into restoration quality [15,16,17,18]. Micro-computed tomography (micro-CT) has emerged as a non-destructive imaging technique for assessing internal adaptation three-dimensionally without sectioning the specimens, yet studies using this methodology in primary teeth remain scarce [15,16,18].

To the best of our knowledge, no previous study has evaluated the internal adaptation of G2-BOND Universal, a two-step universal adhesive, and G-Premio Bond, a one-step universal adhesive, applied using self-etch and selective enamel etching strategies in Class V composite restorations of primary second molars using micro-CT. Addressing this evidence gap is relevant because differences among specific adhesive product–application protocol combinations may be reflected in restoration integrity and long-term clinical performance.

Therefore, the aim of the current in vitro investigation was to compare the whole-cavity interfacial gap volume percentages of four prespecified adhesive system–application protocol combinations in Class V composite restorations placed in primary second molars and evaluated using micro-CT. The null hypothesis was that no statistically significant difference would be observed among the four experimental groups.

## 2. Materials and Methods

Internal adaptation of resin-based posterior composite restorations was examined in vitro using micro-CT across different universal adhesive products and application protocols.

### 2.1. Ethical Approval

Ethical clearance was granted by the Non-Clinical Scientific Research Ethics Committee of the Faculty of Dentistry, Ankara University (Approval No. 15/1; 3 November 2025). The study used extracted human primary molars, and written informed consent for tooth collection and research use was obtained from the parents or legal guardians before collection. All procedures complied with the ethical principles of the World Medical Association Declaration of Helsinki, including those applicable to research involving identifiable human biological material and data [19].

### 2.2. Study Design and Reporting

The investigation used a randomized in vitro experimental framework incorporating simple random allocation, blinded outcome assessment, and prespecified measures to reduce potential bias. The design and reporting followed the Checklist for Reporting In-vitro Studies (CRIS) [20]. Applicable CRIS items were addressed during sample-size planning, specimen allocation and preparation, implementation of the experimental protocol, outcome evaluation, and statistical analysis.

### 2.3. Sample Size Calculation

The sample size was determined a priori using G*Power software (Version 3.1.9.7; Heinrich Heine University, Düsseldorf, Germany). Because the prespecified primary objective was an omnibus comparison among four independent adhesive system–application protocol groups, the calculation was based on a one-way fixed-effects ANOVA with four groups. The calculation was performed before data collection, when the distribution of the outcome variable was unknown. In the absence of pilot data or a sufficiently comparable previous micro-CT investigation in primary teeth from which a data-driven effect size could be derived, an effect size of f = 0.40 was selected, corresponding to the conventional threshold for a large omnibus effect proposed by Cohen [21]. Setting α at 0.05 and the target statistical power at 80% yielded a required total of 76 specimens, corresponding to 19 specimens per group. The achieved power was 82.3%, with a noncentrality parameter (λ) of 12.16, a critical F value of 2.73, and numerator and denominator degrees of freedom of 3 and 72, respectively. This calculation was intended to detect an overall difference among the four experimental groups and was not designed to determine the power of an adhesive system × application mode interaction.

### 2.4. Specimen Collection and Storage

The study included 76 extracted human maxillary and mandibular primary second molars obtained from the Departments of Pediatric Dentistry and Oral and Maxillofacial Surgery at Ankara University Faculty of Dentistry. Tooth collection proceeded after ethics approval and receipt of written informed consent from each child’s parent or legal guardian. Because every specimen originated from a different child, no participant contributed more than one tooth, and participant-level clustering was absent. The donors were 5–8 years of age and comprised 46 male and 30 female children. Donor age and sex were recorded only for the overall study sample and were not available according to experimental group. The inclusion criteria comprised teeth free from caries, restorations, fractures, and cracks. In addition, only teeth exhibiting physiological root resorption involving no more than one-third of the root length from the furcation area were included. This criterion was applied consistently to all specimens; however, the precise degree of physiological root resorption was not recorded as a continuous or categorical specimen-level variable. All specimens were examined under a stereomicroscope (Leica MZ21; Leica Microsystems GmbH, Wetzlar, Germany), and teeth that did not meet the inclusion criteria were excluded from the study. The included teeth had been extracted for clinical indications, including orthodontic reasons or physiological exfoliation associated with more than two-thirds root development of the underlying permanent successor. Immediately after extraction, all tooth surfaces were rinsed under running water to remove blood and soft tissue remnants. The specimens were stored in 0.5% chloramine-T solution for a maximum of eight days between extraction and restoration. Individual storage durations were not standardized; however, all specimens were collected before random allocation to the experimental groups.

### 2.5. Cavity Preparation

**Figure 1:**
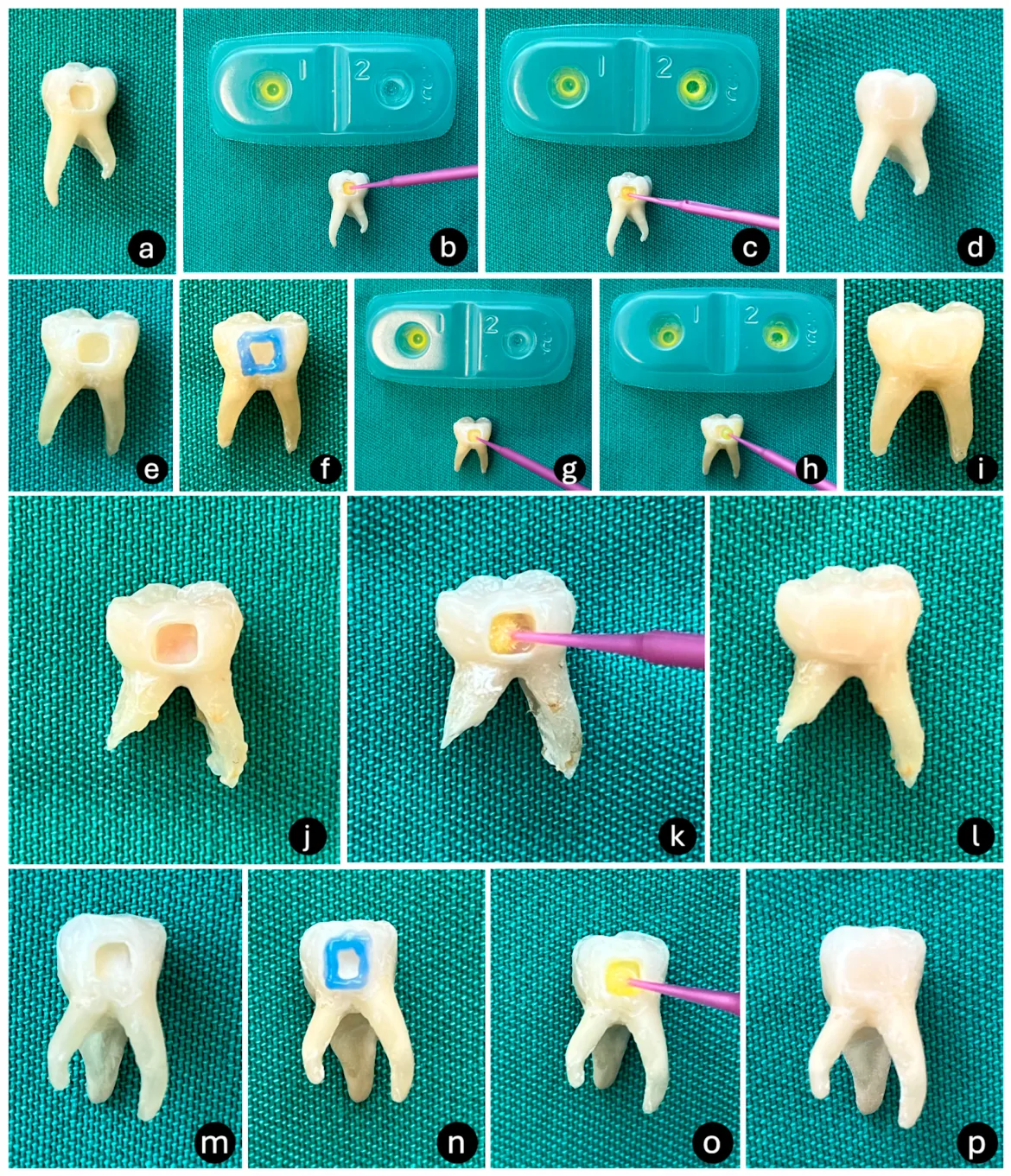
Representative macroscopic photographs illustrating selected stages of cavity preparation, adhesive application, and the completed restoration in each experimental group. (**a**–**d**) Group 1: prepared Class V cavity, application of G2-BOND Universal 1-PRIMER, application of 2-BOND, and completed restoration; (**e**–**i**) Group 2: prepared Class V cavity, selective enamel etching, application of 1-PRIMER, application of 2-BOND, and completed restoration; (**j**–**l**) Group 3: prepared Class V cavity, application of G-Premio Bond, and completed restoration; and (**m**–**p**) Group 4: prepared Class V cavity, selective enamel etching, application of G-Premio Bond, and completed restoration.

After removal from the 0.5% chloramine-T solution, the specimens were rinsed for 5 s using an air–water spray. Residual debris or soft tissue was removed with a periodontal curette when necessary. A standardized Class V preparation was created on the buccal surface of each primary second molar using a high-speed handpiece and a diamond fissure bur (141H010; Meisinger, Neuss, Germany) under continuous water cooling. To maintain consistent cutting performance, the bur was replaced after every fourth preparation. The target cavity dimensions were 3 mm mesiodistally, 3 mm occlusogingivally, and 2 mm in depth. The cavity dimensions were carefully verified at multiple points using the millimeter markings of a periodontal probe by the same operator. Cavities that did not meet the predetermined dimensions were corrected or excluded. The preparation dimensions and cavity volumes were not subsequently confirmed by micro-CT or another measurement method. In addition, the gingival cavity margin was positioned at least 1 mm coronal to the cementoenamel junction. Following cavity preparation, the cavities were rinsed with an air–water spray for 5 s and then dried with oil-free air according to the adhesive manufacturers’ instructions [18]. All cavity preparations and restorative procedures were performed by a single operator (E.A.D.), a pediatric dentistry research assistant with three years of clinical and academic training, using standardized preparation and restorative protocols (Figure 1).

### 2.6. Experimental Groups and Adhesive Procedures

The specimens were randomly assigned to four experimental groups (*n* = 19 per group) according to the universal adhesive system and application mode. Two groups received a one-step universal adhesive, whereas the remaining two groups received a two-step universal adhesive consisting of a separate primer and bonding agent. For each adhesive system, one group was treated using the self-etch approach and the other using the selective enamel etching approach. Consequently, four experimental groups were established: one-step self-etch, one-step selective enamel etching, two-step self-etch, and two-step selective enamel etching. The specimens were allocated to the four experimental groups using a simple randomization sequence generated with Research Randomizer online software (https://www.randomizer.org, accessed on 12 March 2026). Randomization was not stratified according to dental arch or tooth type. The randomization sequence was generated, and the specimens were assigned to the experimental groups, by E.A.D. No separate allocation-concealment mechanism was used. Because group allocation was performed after completion of the standardized cavity preparations, the operator became aware of the assigned group immediately before the adhesive procedure. The post-randomization distribution of maxillary and mandibular teeth in each group is presented in Table 1. The adhesive application protocols for each experimental group are described below (Figure 1).

#### 2.6.1. Group 1: Two-Step Universal Adhesive in the Self-Etch Mode (G2-BOND Universal)

Group 1 specimens (*n* = 19) received no preliminary phosphoric acid conditioning. G2-BOND Universal (GC Corporation, Tokyo, Japan) was used according to its self-etch protocol. Using a disposable microbrush, 1-PRIMER was transferred from a single-use mixing well and spread over all cavity surfaces. After a 10-s application period, gentle air was directed onto the primer for 5 s to promote solvent evaporation. A fresh microbrush was then used to apply 2-BOND, which was dispersed with gentle air for 5 s to produce an even adhesive layer. Polymerization was performed for 10 s with an LED curing unit (Elipar S10; 3M ESPE, St. Paul, MN, USA; 1790 mW/cm^2^). The restorative procedure was subsequently completed using the resin-based posterior composite described below (Figure 1).

#### 2.6.2. Group 2: Two-Step Universal Adhesive in the Selective Enamel-Etching Mode (G2-BOND Universal)

A microbrush was used to apply 35% phosphoric acid gel (Scotchbond Universal Etchant; 3M ESPE, St. Paul, MN, USA) exclusively to the enamel margins for 15 s under direct visual control, with care taken to prevent contact with dentin. The cavities were subsequently rinsed with water for 5 s and dried with oil-free air as recommended by the manufacturer. G2-BOND Universal (GC Corporation, Tokyo, Japan) was then used according to its selective enamel-etching protocol. Using a disposable microbrush, 1-PRIMER was transferred from a single-use mixing well and spread over all cavity surfaces. After a 10-s application period, gentle air was directed onto the primer for 5 s to promote solvent evaporation. A fresh microbrush was then used to apply 2-BOND, which was dispersed with gentle air for 5 s to produce an even adhesive layer. Polymerization was performed for 10 s with an LED curing unit (Elipar S10; 3M ESPE, St. Paul, MN, USA; 1790 mW/cm^2^). The restorative procedure was subsequently completed using the resin-based posterior composite described below (Figure 1).

#### 2.6.3. Group 3: One-Step Universal Adhesive in the Self-Etch Mode (G-Premio Bond)

Group 3 specimens (*n* = 19) were not conditioned with phosphoric acid before bonding. G-Premio Bond (GC Corporation, Tokyo, Japan) was used according to its self-etch protocol. The adhesive was transferred to a single-use mixing well and delivered with a disposable microbrush to coat all cavity surfaces uniformly. After remaining on the cavity surfaces for 10 s, the adhesive was initially exposed to gentle air, followed by a strong oil-free air stream for approximately 5 s to promote solvent evaporation and obtain a thin adhesive layer. The adhesive was polymerized for 10 s with an LED curing unit (Elipar S10; 3M ESPE, St. Paul, MN, USA; 1790 mW/cm^2^). The restorative procedure was subsequently completed using the resin-based posterior composite described below (Figure 1).

#### 2.6.4. Group 4: One-Step Universal Adhesive in the Selective Enamel-Etching Mode (G-Premio Bond)

A microbrush was used to apply 35% phosphoric acid gel exclusively to the enamel margins for 15 s under direct visual control, with care taken to prevent contact with dentin. The cavities were subsequently rinsed with water for 5 s and dried with oil-free air as recommended by the manufacturer. G-Premio Bond (GC Corporation, Tokyo, Japan) was then used according to its selective enamel-etching protocol. The adhesive was transferred to a single-use mixing well and delivered with a disposable microbrush to coat all cavity surfaces uniformly. After remaining on the cavity surfaces for 10 s, the adhesive was initially exposed to gentle air, followed by a strong oil-free air stream for approximately 5 s to promote solvent evaporation and obtain a thin, uniform adhesive layer. The adhesive was polymerized for 10 s with an LED curing unit (Elipar S10; 3M ESPE, St. Paul, MN, USA; 1790 mW/cm^2^). The restorative procedure was subsequently completed using the resin-based posterior composite described below (Figure 1).

### 2.7. Restorative Procedure

Restorations were completed with a resin-based posterior composite (G-ænial Posterior; GC Corporation, Tokyo, Japan). Each cavity was filled to the standardized margins in a single increment, and the material was adapted to the cavity walls by the same operator (E.A.D.) using an identical plastic filling instrument and a consistent manual technique. Neither composite volume nor insertion pressure was measured instrumentally. During all adhesive and composite polymerization procedures, the light-guide tip was maintained perpendicular to the restoration surface at an approximate distance of 1 mm. The output of the curing unit was checked with a Woodpecker LED Light Meter (LM-1; Guilin Woodpecker Medical Instrument Co., Ltd., Guilin, China). The composite was exposed to the LED curing unit for 10 s (Elipar S10; 3M ESPE, St. Paul, MN, USA; 1790 mW/cm^2^). After curing, the same operator finished and polished every restoration using Sof-Lex™ Finishing and Polishing Discs (3M ESPE, St. Paul, MN, USA) in accordance with the manufacturer’s instructions (Figure 1).

### 2.8. Artificial Aging

Following completion of the restorative procedures, all specimens were stored in distilled water at 37 °C for 24 h and subsequently subjected to artificial aging by thermocycling to simulate intraoral temperature fluctuations. Thermocycling was performed using a thermocycling device (SD Mechatronik GmbH, Feldkirchen-Westerham, Germany) for 5000 cycles between 5 °C and 55 °C, with a 30-s dwell time in each bath. Thermocycling was used as a standardized laboratory hydrothermal aging procedure and was not considered equivalent to a specific duration of clinical service [22,23,24]. After thermocycling, the internal adaptation of all restorations was evaluated using micro-CT. Before image analysis, all micro-CT datasets were assigned random numerical codes by A.D. The coded datasets were subsequently analyzed by A.B., who had no access to the group assignments during image analysis and measurement procedures.

### 2.9. Micro-CT Analysis

After completion of the restorative and artificial-aging procedures, the specimens underwent micro-CT scanning to assess restoration internal adaptation (SkyScan 1275; Bruker Micro-CT, Kontich, Belgium). The X-ray source was operated at 80 kV and 125 μA and image acquisition was performed with a 1-mm aluminum filter and an isotropic voxel size of 20 μm, in accordance with previously reported protocols [16,25]. Each specimen was rotated through 360° at angular increments of 0.2°. Projection data were reconstructed in NRecon software (version 1.7.4.2; Bruker Micro-CT, Kontich, Belgium) using a smoothing value of 3, ring artifact correction of 7, and beam-hardening correction of 38% [26]. Regions of interest (ROIs) were delineated in CTAn software (version 1.23.0.2; Bruker Micro-CT, Kontich, Belgium) [25,26]. A volume of interest (VOI) covering the complete prepared cavity and incorporating both enamel and dentin interfaces was then established for quantitative assessment [27]. Because the prespecified outcome represented the whole-cavity interfacial gap volume percentage, separate analytical regions were not created for the enamel interface, dentin interface, or individual cavity walls. Three-dimensional renderings of the reconstructed datasets were generated using CTVox software (version 1.23.0.2; Bruker Micro-CT, Kontich, Belgium).

### 2.10. Internal Adaptation Analysis and Blinded Assessment

Micro-CT analysis was performed in CTAn software (version 1.23.0.2; Bruker Micro-CT, Kontich, Belgium) using a fixed global-thresholding protocol. Before thresholding, the analysis was spatially restricted to predefined ROIs/VOIs encompassing the restoration and its associated interfacial gap region. Thresholding was therefore not applied to the entire tooth volume or used to classify the surrounding enamel and dentin as separate material classes. Within these predefined VOIs, grayscale values of 110–255 were used to segment the restorative material, whereas values of 1–109 were used to identify voids. These operational threshold ranges were applied uniformly to all reconstructed datasets, and specimen-specific adjustments were not permitted. Mineralized tooth structures and voids outside the predefined restoration–cavity region were excluded from all volumetric calculations. Marginal defects communicating with the external environment were included when they were located within the predefined cavity VOI. Total cavity volume (TCV) was defined as the volume of the predefined cavity VOI. Total void volume (TVV) represented the total segmented void volume within this VOI, whereas internal composite void volume (IVV) represented voids located within the restorative material. For IVV measurement, the initial cavity VOI was spatially reduced to encompass the restoration volume and its internal void regions, and voids within this reduced VOI were quantified separately. Interfacial gap volume (IGV) was calculated as follows:

*IGV* = *TVV* − *IVV*

The whole-cavity interfacial gap volume percentage was calculated using the following equation:

*Whole-cavity interfacial gap volume percentage* (%) = (*IGV*/*TCV*) × 100

**Figure 2:**
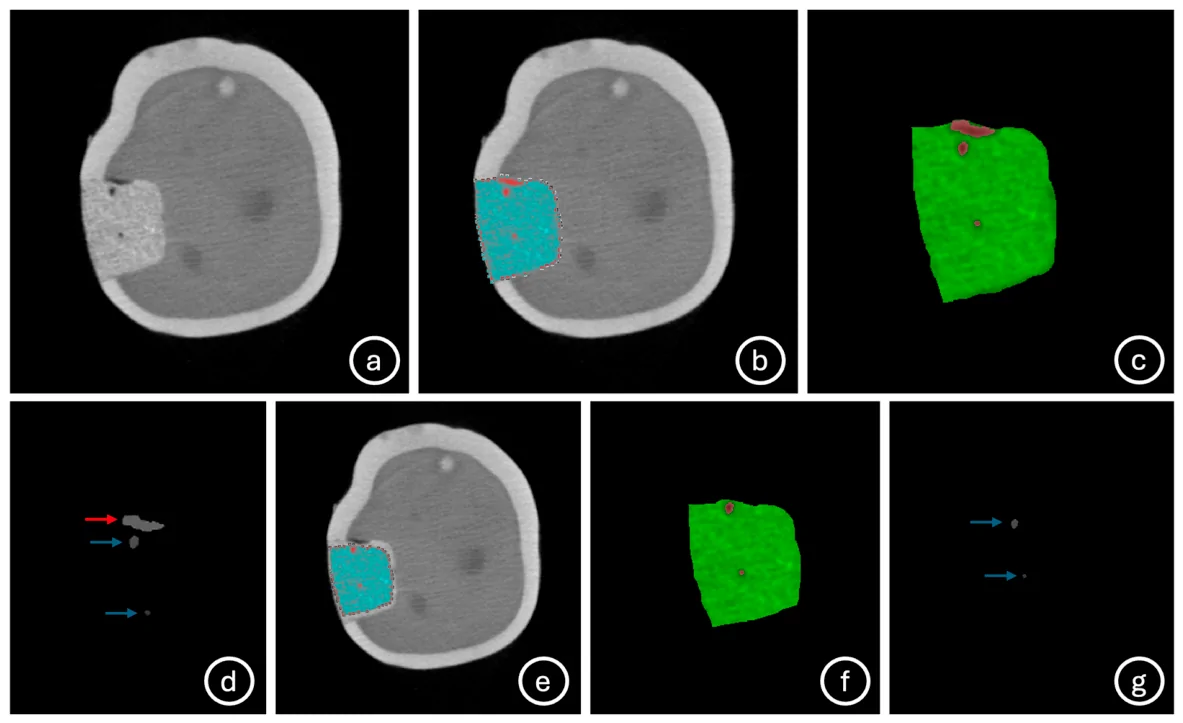
Representative micro-CT images illustrating the segmentation and volumetric workflow used to calculate the whole-cavity interfacial gap volume percentage. (**a**) Original reconstructed micro-CT image showing the tooth structure and restorative material. (**b**) Delineation of the region of interest (ROI) used to define the complete cavity volume of interest (VOI) within the predefined spatial boundaries. (**c**) Segmentation of the total void volume within the cavity VOI. (**d**) Identification of voids located within the restorative material (blue arrows) and interfacial gaps at the restoration–tooth interface (red arrows). (**e**) Spatial reduction in the cavity VOI to define a restoration-specific VOI. (**f**,**g**) Segmentation and quantification of voids located within the restorative material. Thresholding was applied only within the spatially predefined VOIs and not to the entire tooth volume.

This outcome combined gaps associated with both enamel and dentin interfaces and was not subdivided according to dental tissue or individual cavity wall. All micro-CT analyses were performed by a single evaluator (A.B.) with more than 15 years of experience in micro-CT imaging and analysis. The evaluator was blinded to the experimental group allocation and applied the same predefined segmentation criteria to all specimens. The segmentation and volumetric quantification workflow is illustrated in Figure 2.

**Figure 3:**
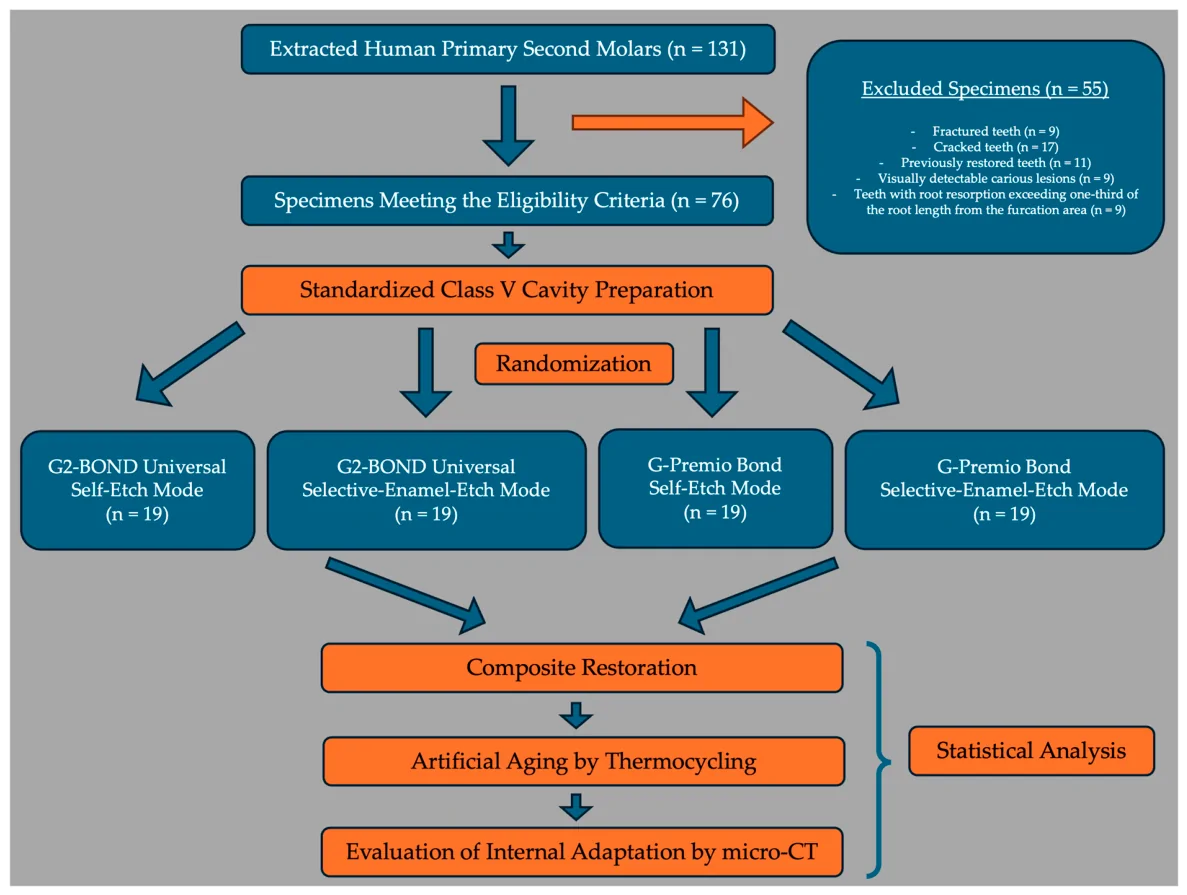
Methodological flowchart illustrating specimen selection and randomization, experimental procedures, artificial aging by thermocycling, micro-CT evaluation of internal adaptation and statistical analysis.

The overall experimental workflow, including specimen collection, eligibility assessment, randomization, experimental procedures, artificial aging, micro-CT analysis, and statistical evaluation, is illustrated in Figure 3.

### 2.11. Statistical Analysis

The primary statistical analyses were performed using IBM SPSS Statistics for Windows (Version 30.0; IBM Corp., Armonk, NY, USA). The data were summarized using the mean, standard deviation (SD), median, first quartile (Q1), third quartile (Q3), interquartile range (IQR), and extreme values. Distributional normality was examined with the Shapiro–Wilk test. Because at least some experimental groups departed from normality, the four independent groups were compared using the Kruskal–Wallis test. The Kruskal–Wallis epsilon-squared effect size was calculated as ε^2^ = (H − k + 1)/(N − k), where H represents the Kruskal–Wallis test statistic, k the number of groups, and N the total sample size. A significant omnibus result was followed by Dunn pairwise comparisons with Bonferroni correction for multiple testing. Statistical significance was defined as *p* < 0.05. For the single pairwise comparison that remained statistically significant after Dunn–Bonferroni adjustment, Cliff’s delta (δ) was calculated as a nonparametric effect-size measure. A negative δ value indicated lower observations in the first-listed group. The 95% confidence interval was estimated using bias-corrected and accelerated bootstrap resampling with 50,000 resamples. Cliff’s delta and its confidence interval were calculated using Python 3.12.13 and SciPy 1.17.0.

## 3. Results

**Figure 4:**
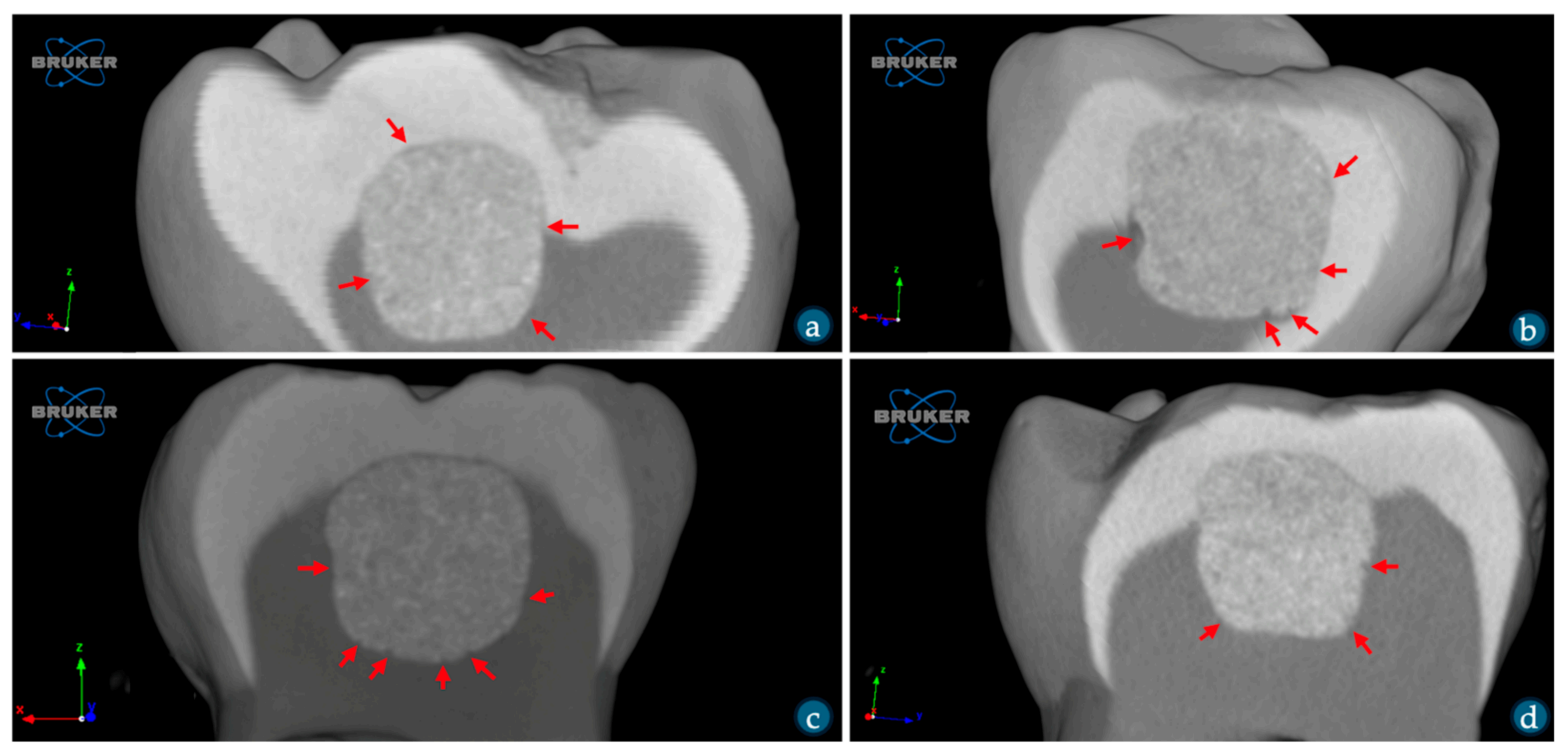
Representative three-dimensional volume-rendered micro-CT views illustrating the overall spatial relationship between the restoration and surrounding tooth structure. The specimens are displayed from different viewing orientations to allow visualization of the restoration–tooth interface, and the red arrows indicate illustrative interfacial defects. (**a**) Group 1, G2-BOND Universal/self-etch; (**b**) Group 2, G2-BOND Universal/selective enamel etching; (**c**) Group 3, G-Premio Bond/self-etch; and (**d**) Group 4, G-Premio Bond/selective enamel etching. These images are provided for illustrative purposes only and should not be interpreted as visual evidence of differences among the experimental groups.

**Figure 5:**
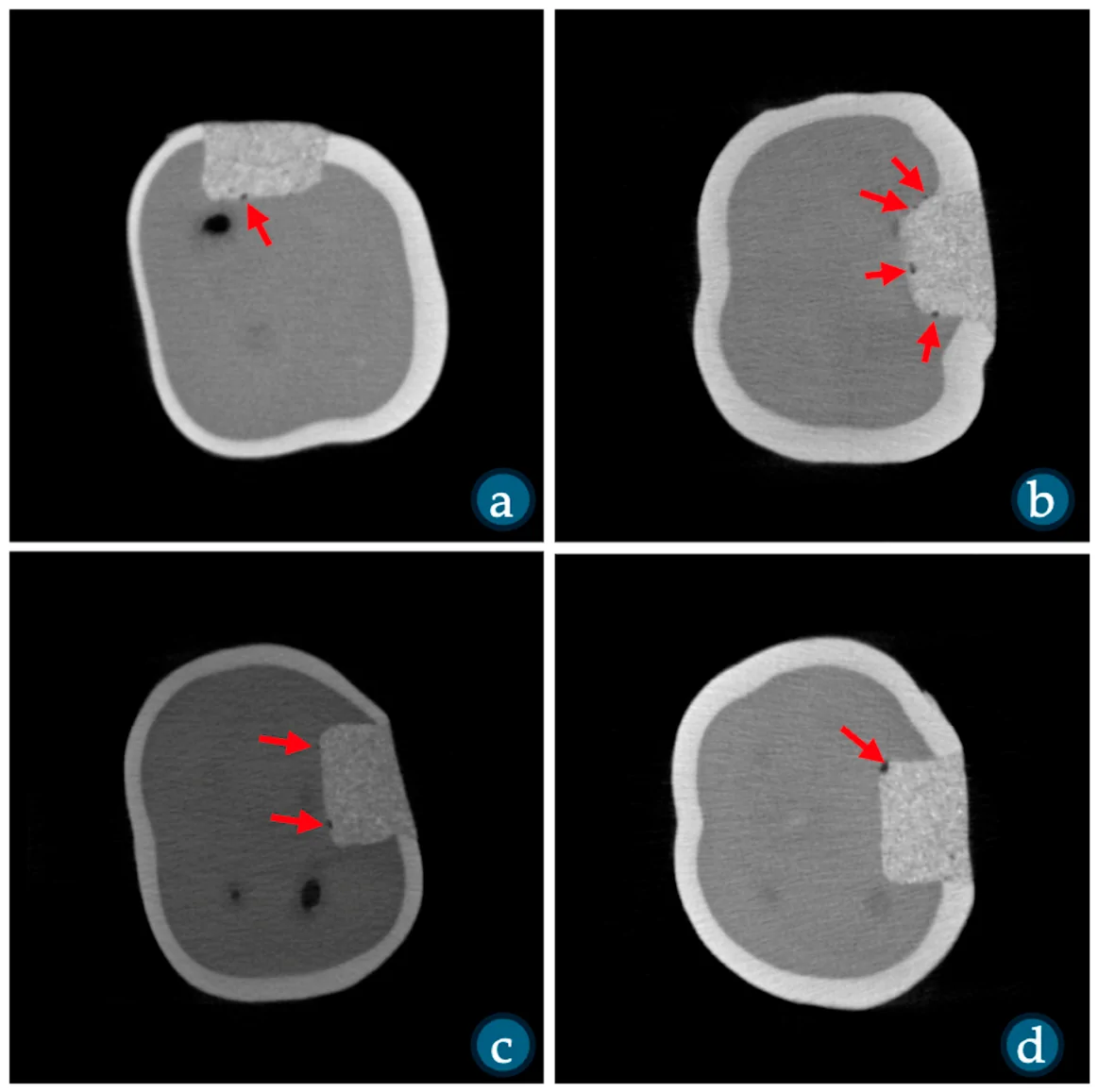
Representative axial two-dimensional micro-CT sections, oriented perpendicular to the long axis of the tooth, providing higher-magnification visualization of localized restoration–tooth interfacial defects. Red arrows indicate illustrative gaps between the composite restoration and cavity walls. (**a**) Group 1, G2-BOND Universal/self-etch; (**b**) Group 2, G2-BOND Universal/selective enamel etching; (**c**) Group 3, G-Premio Bond/self-etch; and (**d**) Group 4, G-Premio Bond/selective enamel etching. These images are provided for illustrative purposes only and should not be interpreted as visual evidence of differences among the experimental groups.

A total of 76 specimens were included in the study and randomly allocated to four independent experimental groups according to the universal adhesive system and application mode (*n* = 19 per group). Specimens restored using G2-BOND Universal were assigned to the self-etch (Group 1) and selective enamel etching (Group 2) groups, whereas specimens restored using G-Premio Bond were assigned to the self-etch (Group 3) and selective enamel etching (Group 4) groups (Figure 4 and Figure 5). The distribution of the experimental groups is presented in Table 1. Each specimen was obtained from a different donor, and no participant contributed more than one tooth. The study population consisted of children aged 5–8 years, including 46 male and 30 female donors. The distribution of maxillary and mandibular teeth was 11/8 in Group 1, 9/10 in Group 2, 8/11 in Group 3, and 9/10 in Group 4, respectively (Table 1).

**Figure 6:**
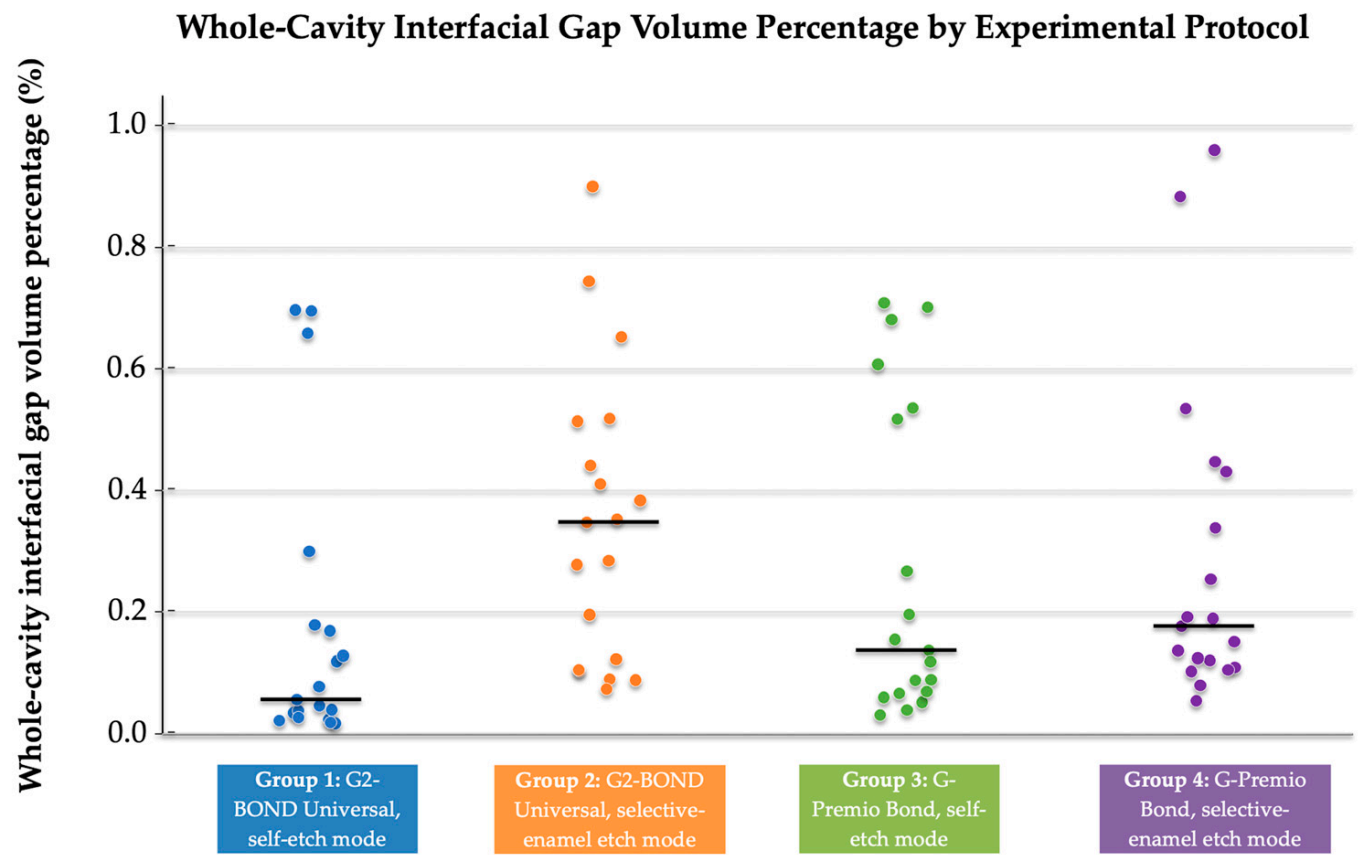
Individual whole-cavity interfacial gap volume percentage measurements across the four experimental protocol groups. Each point represents one specimen, and the horizontal black lines indicate the group medians.

Descriptive statistics for the whole-cavity interfacial gap volume percentage of the four experimental groups are presented in Table 2. All values in Table 2 are expressed as percentages of the total cavity volume; for example, a value of 0.177 represents 0.177%. The mean whole-cavity interfacial gap volume percentage ranged from 0.177 to 0.348 across the groups. The lowest mean value was observed in Group 1 (G2-BOND Universal, self-etch mode), whereas the highest mean value was recorded in Group 2 (G2-BOND Universal, selective enamel-etching mode). Similarly, the median value was lowest in Group 1 and highest in Group 2. The minimum and maximum values were 0.018–0.698 for Group 1, 0.074–0.900 for Group 2, 0.031–0.709 for Group 3, and 0.054–0.960 for Group 4 (Table 2 and Figure 6). Median whole-cavity interfacial gap volume percentages were 0.057% (Q1–Q3: 0.031–0.174) in Group 1, 0.348% (0.114–0.478) in Group 2, 0.137% (0.069–0.527) in Group 3, and 0.177% (0.115–0.385) in Group 4 (Table 2). The distributions of the individual measurements and the corresponding group medians are shown in Figure 6.

Shapiro–Wilk test results are summarized in Table 3. Significant departures from normality were identified for Group 1 (W = 0.666, *p* < 0.001), Group 3 (W = 0.786, *p* = 0.001), and Group 4 (W = 0.763, *p* < 0.001). Group 2 showed no statistically significant departure from a normal distribution (W = 0.917, *p* = 0.102). Because the normality criterion was not satisfied across all four groups, subsequent between-group comparisons were conducted using nonparametric methods.

Because the normality assumption was not satisfied, whole-cavity interfacial gap volume percentages were compared using the Kruskal–Wallis test. An overall group difference was detected (χ^2^ = 10.329, df = 3, *p* = 0.016; ε^2^ = 0.102; Table 4).

Dunn–Bonferroni pairwise results are presented in Table 5. Only the comparison between Group 1 and Group 2 remained statistically significant after multiplicity adjustment (mean rank difference = −21.947; unadjusted *p* = 0.002; adjusted *p* = 0.013). The mean whole-cavity interfacial gap volume percentages were 0.177% and 0.348% in Groups 1 and 2, respectively, corresponding to an absolute difference of 0.171 percentage points. The respective medians were 0.057% and 0.348%, corresponding to an absolute difference of 0.291 percentage points. Cliff’s delta was −0.535 (95% BCa bootstrap CI: −0.795 to −0.136), indicating a tendency toward lower observations in Group 1. No other comparison was statistically significant: the adjusted *p* values were 0.538 for Group 1 versus Group 3, 0.108 for Group 1 versus Group 4, and 1.000 for Group 2 versus Group 3, Group 2 versus Group 4, and Group 3 versus Group 4 (Table 5).

## 4. Discussion

Dental caries remains one of the most prevalent chronic diseases of childhood and represents a major public health concern [28,29]. Resin composites are widely used restorative materials in primary teeth because of their esthetic properties and compatibility with minimally invasive approaches. However, polymerization shrinkage may compromise the restoration–tooth interface and contribute to gap formation, marginal deterioration, and microleakage [15,16,17,18]. Universal adhesives permit different application strategies, including self-etch and selective enamel etching, and may simplify restorative procedures in pediatric patients [1,2,9,10,11,30,31,32]. Because primary enamel and dentin differ structurally from their permanent counterparts, evidence obtained from permanent teeth cannot be directly extrapolated to primary dentition [3,7,13,14,18]. Moreover, most previous studies have used bond-strength outcomes, whereas micro-CT provides a non-destructive three-dimensional assessment of interfacial gap formation [1,9,10,11,12,15,16,18].

The findings of the present study should be interpreted within the context of its in vitro design. Although in vitro studies provide important advantages by allowing standardization of specimens, cavity preparation and experimental procedures under controlled conditions, they cannot fully reproduce the complex biological and mechanical environment of the oral cavity [33,34]. To improve the clinical relevance of the experimental model, all specimens were subjected to 5000 thermal cycles between 5 °C and 55 °C, simulating hydrothermal aging before micro-CT evaluation [18,35]. This procedure should not be interpreted as reproducing a specific duration of intraoral service. Thermal aging may affect the adhesive interface through repeated thermal stresses and hydrolytic degradation, with the magnitude of these effects potentially varying among specific adhesive product–application protocol combinations [18,35,36,37,38]. Therefore, while the present findings should not be directly extrapolated to long-term clinical performance, they provide laboratory-based information regarding differences in three-dimensional internal adaptation among the four tested product–application protocol combinations.

The primary analysis identified an overall difference in whole-cavity interfacial gap volume percentage among the four experimental groups. Accordingly, the null hypothesis of no statistically significant difference among the four experimental groups was rejected at the omnibus level. Dunn–Bonferroni-adjusted pairwise comparisons showed that the G2-BOND Universal self-etch group had a lower whole-cavity interfacial gap volume percentage than the corresponding selective enamel-etching group. No other adjusted pairwise comparison reached statistical significance. Because the study did not establish a statistically supported adhesive system × application mode interaction, the significant product-specific contrast should not be interpreted as evidence that the effect of the application mode differed between the two adhesive systems. Accordingly, the interpretation of the findings was restricted to the observed difference between the two G2-BOND Universal protocol groups. The absolute difference between these groups was 0.171 percentage points based on the means and 0.291 percentage points based on the medians. The corresponding Cliff’s delta was −0.535, although its confidence interval indicated uncertainty regarding the precision of the estimated effect. Importantly, no clinically meaningful threshold has been established for whole-cavity interfacial gap volume percentage. Therefore, the observed statistical difference and effect-size estimate should not be interpreted as demonstrating clinically meaningful superiority or predicting differences in restoration longevity.

The present findings should not be interpreted as conflicting with previous studies reporting improved enamel bond strength after phosphoric acid etching because those studies evaluated localized enamel bond strength, whereas the present investigation quantified a combined whole-cavity outcome incorporating both enamel and dentin interfaces. Primary enamel commonly presents a superficial aprismatic layer, which may influence its response to phosphoric acid conditioning and has historically led to recommendations for longer etching times. In the present study, cavity preparation with a diamond bur was expected to disrupt this superficial layer; therefore, the findings should be interpreted specifically within the 15-s selective enamel etching protocol used. For example, Antoniazzi et al. [39] demonstrated superior enamel bond strength with the etch-and-rinse approach, while the systematic review by Da Rosa et al. [11] concluded that phosphoric acid conditioning mainly benefits enamel bonding. Accordingly, the enamel bond-strength advantages reported in those studies cannot be directly compared with the whole-cavity micro-CT outcome evaluated here. Localized bond-strength measurements and volumetric assessment of interfacial gap formation represent distinct but complementary aspects of adhesive performance, and improvement in one outcome does not necessarily imply a corresponding improvement in the other. Conversely, the present whole-cavity measurement cannot determine whether selective enamel etching produced an enamel-specific effect. Any change confined to the enamel interface may have been diluted or obscured by the combined assessment of the enamel and dentin interfaces and the other cavity regions.

The mechanism underlying the observed difference between the two G2-BOND Universal protocols cannot be determined from the present findings. Both groups used the same two-step adhesive system, including the same primer and hydrophobic bonding layer; therefore, adhesive architecture cannot explain the intraproduct contrast. The only experimental difference between these groups was the use or omission of selective phosphoric acid conditioning of the enamel margins. Previous studies comparing G2-BOND Universal with G-Premio Bond or discussing broader differences between one-step and two-step adhesives [40,41] involved different product comparisons and outcomes and therefore cannot directly explain the present intraproduct finding. Similarly, investigations of marginal adaptation or other adhesive product–application protocol combinations [42,43] provide only indirect contextual evidence. Because enamel- and dentin-specific gap distributions, hybrid-layer morphology, resin infiltration, water sorption, and chemical degradation were not evaluated, explanations based on these mechanisms would remain hypothesis-generating. Accordingly, interpretation should be restricted to the observation that, under the specified laboratory conditions, the G2-BOND Universal self-etch protocol produced a lower measured whole-cavity interfacial gap volume percentage than the corresponding selective enamel-etching protocol after adjustment for multiple comparisons.

The present study has several limitations that should be considered when interpreting the findings. This was an in vitro investigation, which allowed standardization of specimen selection, cavity preparation and restorative procedures but could not fully reproduce the biological and mechanical complexity of the oral environment. Although thermocycling provided standardized laboratory hydrothermal aging, it did not reproduce a specific duration or the full complexity of intraoral service. Mechanical loading, pulpal pressure, salivary exposure, biofilm challenge, parafunctional forces, and long-term mechanical fatigue were not included. Therefore, the findings should not be directly extrapolated to clinical longevity or long-term intraoral performance.

In this study, only extracted primary second molars restored with standardized Class V cavities were included. The box-shaped Class V cavities used in this study had a high C-factor, and placement of the composite in a single increment may have increased polymerization-shrinkage stress and contributed to gap formation. Incremental placement, flowable liners, or transparent cervical matrices may produce different outcomes; therefore, the standardized 3 × 3 × 2 mm preparation should be regarded as an experimental model rather than a recommended clinical technique. Despite random allocation, inherent variations in dentin thickness, mineralization, tubular density and other substrate-related characteristics may have influenced adhesive performance. Although each tooth was obtained from a different donor and participant-level clustering was therefore not present, donor age and sex were available only for the overall study sample and could not be described according to experimental group. Furthermore, the precise degree of physiological root resorption was not quantified for each specimen beyond the standardized eligibility criterion. Consequently, the comparability of the groups with respect to these donor- and specimen-level characteristics could not be formally evaluated. In addition, individual storage durations were not standardized, and cavity dimensions, composite volume, and insertion pressure were not verified using instrument-based quantitative methods, although all procedures were performed by the same operator using a standardized protocol.

On the other hand, only two universal adhesive systems and two application strategies were evaluated; therefore, the findings cannot be generalized to all universal adhesives with different chemical compositions, functional monomers or solvent systems. In addition, restoration performance was assessed exclusively by three-dimensional internal adaptation using micro-CT. An additional major limitation concerns the anatomical specificity of the outcome measurement. Selective enamel etching is intended primarily to modify the enamel component of the adhesive interface; however, the outcome evaluated in this study combined interfacial gaps across the entire cavity, including both enamel and dentin regions. Because separate masks for the enamel and dentin interfaces and individual cavity walls were not prospectively generated during segmentation, a reliable regional analysis could not be performed within the predefined analytical workflow. Therefore, an enamel-specific effect may have been diluted or obscured by the whole-cavity measurement. The present findings should not be interpreted as evidence regarding enamel bonding or the overall clinical performance of the tested adhesives. Although micro-CT provides a non-destructive volumetric assessment of the restoration–tooth interface, it does not evaluate other clinically relevant parameters such as bond strength, marginal adaptation, microleakage, hybrid-layer morphology, or nanoleakage.

Furthermore, the sample size calculation was based on an assumed large omnibus effect for the comparison of four independent protocol groups and was not designed to detect smaller effects or an adhesive system × application mode interaction. Therefore, pairwise comparisons that did not reach statistical significance should not be interpreted as evidence of equivalence between the groups. Accordingly, the present findings should be interpreted as laboratory-based evidence and should not be considered direct predictors of long-term clinical performance.

The 20 μm isotropic voxel size represented the lower spatial detection limit of the micro-CT protocol; therefore, gaps smaller than this resolution may not have been reliably identified. Although fixed threshold ranges and predefined segmentation criteria were applied uniformly by a single blinded evaluator, the operational threshold values and segmentation procedure were not independently validated against a reference standard. A separate histogram-based calibration, formal intraobserver repeatability assessment, and threshold-sensitivity analysis were not performed. Consequently, measurement error and the robustness of the calculated gap percentages to small changes in the selected threshold limits could not be quantified, which limits the precision of the between-group comparison. This limitation is particularly relevant given the low measured gap percentages and the 20 μm voxel size. Partial-volume effects and overlapping attenuation values may also have affected the identification and quantification of very small interfacial defects. Accordingly, the statistically significant product-specific contrast should be interpreted specifically within the predefined segmentation protocol used in this study. Finally, because micro-CT scanning was performed only after thermocycling, gaps created during restoration placement could not be distinguished from those developing or enlarging during thermal aging.

Future studies should evaluate a broader range of universal adhesive systems with different chemical compositions and application strategies under more clinically relevant conditions. Future micro-CT studies should also prospectively define and analyze the enamel and dentin interfaces and individual cavity regions separately to determine whether selective enamel etching produces anatomically localized effects that may not be detectable using a whole-cavity outcome. Combining micro-CT with complementary assessment methods, including bond strength testing, marginal adaptation, microleakage analysis and microscopic evaluation of the adhesive interface, would provide a more comprehensive understanding of adhesive performance. Furthermore, longer-term thermomechanical aging protocols and well-designed prospective randomized clinical trials are required to determine whether differences in measured whole-cavity interfacial gap volume percentage among specific protocols are associated with clinically meaningful differences in the longevity of composite restorations in primary teeth. In addition, future micro-CT investigations should incorporate prespecified intraobserver repeatability testing, measurement-error estimation, and threshold-sensitivity analyses to establish the robustness of volumetric gap measurements.

## 5. Conclusions

Within the limitations of this in vitro study, an overall difference in whole-cavity interfacial gap volume percentage was detected among the four tested adhesive system–application protocol combinations. Following adjustment for multiple comparisons, the G2-BOND Universal self-etch group showed a lower whole-cavity interfacial gap volume percentage than the corresponding G2-BOND Universal selective enamel-etching group. No other adjusted pairwise comparison reached statistical significance. These product-specific laboratory findings do not establish a general advantage of the self-etch strategy over selective enamel etching, demonstrate equivalence between the other experimental groups, or support an interaction between adhesive system and application mode.

## Figures and Tables

**Table 1:** Distribution of the specimens according to experimental group, application mode, adhesive system, and dental arch.

Groups	Application Mode	Adhesive System	Maxillary, *n*	Mandibular, *n*	Total, *n*
Group 1	Self-etch	G2-BOND Universal	11	8	19
Group 2	Selective Enamel Etching	G2-BOND Universal	9	10	19
Group 3	Self-etch	G-Premio Bond	8	11	19
Group 4	Selective Enamel Etching	G-Premio Bond	9	10	19
Total			37	39	76

**Table 2:** Descriptive statistics of the whole-cavity interfacial gap volume percentage according to the experimental groups.

Groups	*n*	Mean (%)	Standard Deviation (%)	Median (%)	Minimum (%)	Maximum (%)	Q1–Q3 (%)	IQR (%)
Group 1: G2-BOND Universal, self-etch mode	19	0.177	0.237	0.057	0.018	0.698	0.031–0.174	0.143
Group 2: G2-BOND Universal, selective enamel-etching mode	19	0.348	0.240	0.348	0.074	0.900	0.114–0.478	0.364
Group 3: G-Premio Bond, self-etch mode	19	0.270	0.258	0.137	0.031	0.709	0.069–0.527	0.458
Group 4: G-Premio Bond, selective enamel-etching mode	19	0.284	0.263	0.177	0.054	0.960	0.115–0.385	0.270

**Table 3:** Results of the Shapiro–Wilk test assessing the normality of the whole-cavity interfacial gap volume percentage in the experimental groups.

Groups	Shapiro–Wilk W	*p*
Group 1: G2-BOND Universal, self-etch mode	0.666	<0.001
Group 2: G2-BOND Universal, selective enamel-etching mode	0.917	0.102
Group 3: G-Premio Bond, self-etch mode	0.786	0.001
Group 4: G-Premio Bond, selective enamel-etching mode	0.763	<0.001

**Table 4:** Comparison of the whole-cavity interfacial gap volume percentage among the experimental groups using the Kruskal–Wallis test.

Test	χ^2^	df	*p*	Effect Size
Kruskal–Wallis	10.329	3	0.016	ε^2^ = 0.102

**Table 5:** Pairwise comparisons of the whole-cavity interfacial gap volume percentage among the experimental groups using the Dunn–Bonferroni post hoc test.

Pairwise Comparisons	Mean Rank Difference	Unadjusted *p* Value	Bonferroni-Adjusted *p* Value
Group 1 vs. Group 2	−21.947	0.002	0.013
Group 1 vs. Group 3	−12.158	0.090	0.538
Group 1 vs. Group 4	−16.947	0.018	0.108
Group 2 vs. Group 3	9.789	0.172	1.000
Group 2 vs. Group 4	5.000	0.485	1.000
Group 3 vs. Group 4	−4.789	0.504	1.000

## Data Availability

The raw data supporting the findings of this study are available from the corresponding author upon reasonable request.

## Abbreviations

The following abbreviations are used in this manuscript:

micro-CT	micro-computed tomography
WMA	World Medical Association
CRIS	Checklist for Reporting In-vitro Studies
ROI	region of interest
VOI	volume of interest
SD	standard deviation
ANOVA	analysis of variance
